# Is home environment associated with child fluid reasoning abilities in middle childhood in high-risk settings? findings from a cross-sectional study in Pakistan

**DOI:** 10.1186/s12887-024-05108-z

**Published:** 2024-10-08

**Authors:** Muneera A. Rasheed, Sondre Aasen Nilsen, Tor A. Strand, Fariha Shaheen, Ingrid Kvestad

**Affiliations:** 1 Centre for International Health, Department of Global Public Health and Primary Care, University of Bergen, Bergen, Norway; 2 Department of Health Promotion and Development, Faculty of Psychology, University of Bergen, Bergen, Norway; 3 Innlandet Hospital Trust, Lillehammer, Norway; 4 Department of Paediatrics & Child Health, The Aga Khan University, Karachi, Pakistan

**Keywords:** Home environment, Fluid reasoning abilities, Learning materials and opportunities, Middle childhood, Responsivity

## Abstract

**Background:**

Evidence from low- and middle-income countries (LMIC) suggests that home environment is associated with early childhood development outcomes. However, studies from LMIC that have examined how the home environment during middle childhood is associated with intellectual abilities are scarce. The objective of the study was to explore the association between different aspects of the home environment at 7–8 years and fluid reasoning abilities in a rural, high-risk cohort in Pakistan.

**Methods:**

We employed a cross-sectional research design to examine 1172 children between 7 and 8 years and their families, utilizing the Home Observation for Measurement of Environment for Middle Childhood (HOME-MC) to evaluate various aspects of the home environment and the Fluid Reasoning Index (FRI) of the Wechsler Intelligence for Children (WISC)-5th edition to assess the fluid reasoning abilities of the children. Multivariable regression analyses were used to examine the association between different components of HOME-MC (scored as indices) and FRI scores.

**Findings:**

Learning materials and opportunities (β = 1.74, 95% CI = 1.15, 2.33) and Responsivity (β = 1.73, 95% CI = 1.07, 2.38) indices had the strongest association with FRI score followed by Family companionship index (β = 1.27, 95% CI = 0.63, 1.90). The eight different indices of the HOME-DC explained 22% of the total variation in FRI scores.

**Conclusion:**

We conclude that concurrent learning opportunities, parental responsivity and family companionship at home are associated with fluid reasoning abilities during middle childhood which is comparable to what has been found in early childhood years.

**Supplementary Information:**

The online version contains supplementary material available at 10.1186/s12887-024-05108-z.

## Introduction

The field of Global Early Childhood Development (ECD) and practice is a sub-field of global health that examines young children’s development in low- and middle-income countries, (LMIC) [1]. A significant amount of evidence from this literature indicates that home environment both in terms of its structure (material aspects) and processes (psychosocial aspects) plays a critical role in the development of cognitive, language and socioemotional skills [1–5]. The structural aspects of home environment include factors such as physical space and size, safety, and being conducive to learning with adequate light and organisation [10]. The process, on the other hand, comprises interactions and experiences with the family within or outside the home through parental responsiveness [6], the availability of learning resources [7], parental involvement [8] and types of activities children engage in [9].

Parenting interventions aimed to enhance the quality of these psychosocial aspects such as parental interactions and stimulation in the first three years of life have consistently found to improve children’s developmental outcomes [11, 12]. Conversely, children who live in households where they are not exposed to similar factors at home are at risk of poor development for example McCoy and colleagues (2017) found that lack of stimulation at home in 3–4-year-old children was associated with poor scores on UNICEF’s Multiple Cluster Indicator Survey’s Early Child Development Index score [13]. Given the significance of the home environment for early developmental outcomes, it can be hypothesized that its influence will continue into the later years of childhood.

Empirical findings have also revealed that stimulation at home and parental responsiveness in early childhood were protective for young children against the effects of early adversities on adolescent intellectual abilities [14]. For instance, a study utilizing data from two cohorts in Brazil and South Africa found that nurturing home environments defined by presence of learning opportunities and responsive caregiving measured at age 4 years had long-term positive associations with adolescent cognition at 18 years [14].

However, studies from Global ECD that have explored the role of home environment on child intellectual abilities in middle childhood are scarce. While the first 1000 days of life are important for brain development, middle childhood also deserves attention for a comprehensive understanding of intellectual development in early childhood especially in disadvantaged global settings, as it is the age when children typically start primary school and start applying these abilities for academic achievement [15]. Intellectual abilities, one of the significant predictors for academic achievement in school, begin to stabilise in middle childhood [16, 17]. This underlines the importance of gaining more knowledge on how the home environment influences child abilities during this age. Success in school, a key pathway to human development, is a primary focus of advocacy for Global ECD interventions [18, 19]. Moreover, neuroscience research identifies middle childhood as a distinct developmental period from the first three years [20]. Examining the concurrent factors like home environment can lead to a better understanding of the influences on different components of intellectual development [16, 21]. These insights can help design interventions based on a life course approach [22].

An important component of intellectual abilities is fluid reasoning defined as the capacity to think critically, solve problems, and adapt to new concepts, independent of acquired knowledge which is fundamental for learning [23]. Fluid reasoning allows children to grasp mathematical concepts, understand abstract ideas, and make connections between various subjects [24]. As students progress through primary school, fluid reasoning helps them analyse complex information. Hence, it can be argued that fluid reasoning is essential for academic success. A recent meta-analysis examining the relations between spatial skills and mathematical performance found that fluid reasoning and verbal skills mediated the relationship between spatial and mathematical skills regardless of student grade-level [25].

The Home Observation for the Measurement of Environment (HOME) Inventory developed in the US, is a tool to measure the home environment [26]. The developers defined home environment as “the quantity and quality of stimulation, support and structure available to a child” [26] which is pivotal for a child’s development and well-being. A scoping review to identify nurturing care outcomes in LMIC for children under 5, found the HOME Inventory to be one of the most frequently used tools to measure responsive caregiving and early learning components [27]. The review also highlighted the tool to be multi-dimensional and encompasses different features of the home environment. Moreover, the HOME Inventory has also been found to be sensitive to detect change in home environment as part of ECD programs and interventions in LMIC. Hence, it was deemed appropriate for use in our study to measure home environment. According to a recent review, the HOME Inventory is best understood as a formative index scale composed of causal indicators that are selected based on theory and research to assess the experiences and conditions connected to the home that are predictive of children’s behaviour and development [26]. In other words, the indicators define the construct being measured and thus contrasts reflective measures where an underlying latent construct is believed to exist.

The SCALE 8 study is a follow-up of a cohort assessed at 2 [28, 29] and 4 years [30] for children’s development, home environment and a range of other predictors. Data from the earlier follow-ups, found the groups with high cognitive and language scores also had a higher home environment score [29, 30]. The cohort can be characterised as high risk for not reaching their potential of intellectual abilities as not many families have access to preschools and only one-third of mothers could read and write when evaluated at 4 years. The current study assessed children’s home environment and fluid reasoning skills when children were 7–8 years of age providing an opportunity to further examine the home environment in middle childhood and its association with concurrent intellectual abilities in a high-risk setting. The research objective of the current study was to identify to what extent the specific features of the home environment in middle childhood (ages 7–8) are associated with current child fluid reasoning abilities in a rural, high-risk cohort in Pakistan.

## Methods

### Setting

This study utilized data from a long-term follow-up of an intervention group in Naushahro Feroze, Sindh, Pakistan [28]. Naushahro Feroze is predominantly a rural district comprising a total population (all ages included) of approximately one million. The district is situated 342 km north of Karachi where the Aga Khan University main campus is located. While farming is the main occupation for men, women are generally not employed given the lack of opportunities for them. However, some women are involved in handicraft. A qualitative study in the district with the study population revealed poor quality of education and preschool services especially in the public sector with parental attitudes and lack of engagement with child’s schooling and education as identified challenges [31]. Similar issues have been reported from other rural settings in the country [32, 33].

## Participants

The trial cohort (*N* = 1381, who were evaluated at 2 years for child outcomes) was contacted for re-enrolment in the current follow-up when the children were seven to eight years old between June and November 2017. The inclusion criteria for re-enrolment between 7 and 8 years were completion of assessments at two years of age, no signs of moderate-to-severe impairment, and living in the Sindh province. Re-enrolment began by holding local community meetings with stakeholders (village leaders, community health workers, primary school teachers) to inform them about the study and to leverage help in re-enrolment followed by tracking subjects through household visits, telephone calls and seeking support from relatives and local stakeholders for subjects who are no longer living in the same residence. Once subjects were identified, the study was explained to the head of the household, caregiver and child for informed consent and assent respectively. Participants provided written consent and could decline participation at any time.

The study received approval from the Ethics Review Committee of the Aga Khan University (Approval no. 4421-Ped-ERC-16) and the Regional Committee for Medical Research Ethics Western Norway (REK West) (Approval no. 124126).

## Measures

### Home environment

The HOME Inventory includes four age-based versions of the Inventory: (a) the Infant-Toddler version (0–3 years), (b) the Early Childhood version (3–6 years), (c) the Middle Childhood version (6–10 years), and (d) the Early Adolescent version (10–15 years) [25]. In the current study, the HOME-Middle Childhood (HOME-MC) version was used to measure home environment when children were between 7 and 8 years of age. This version consists of 59 items allocated to 8 different indices measuring different dimensions of the home environment that facilitate learning; (1) Responsivity: the emotional and verbal sensitivity and responsivity of the parent to the child; (2) Encouragement of maturity: parental expectation for the child to demonstrate socially responsible and mature behaviour and conform to family rules; (3) Emotional climate: acceptance by the parent of negative emotional expressions from the child and also own emotional composure; (4) Learning materials & opportunities: availability to the child of developmentally enhancing materials; (5) Enrichment: the extent to which the parents consciously utilize family and community resources to enrich the development of the child with hobbies, recreation, museums, libraries, trips, etc.; (6) Family companionship: child involvement with the parents in activities providing companionship and mutual enjoyment—shopping ventures, visits to or from relatives, recreation, etc.; (7) Family integration: the availability to the child of a father or father-figure and a life history characterized by a consistent primary family group; and (8) Physical environment: relates to the adequacy of the household environment and its immediate surroundings including safety, space per person, free of noise and cleanliness. Each index has a different number of items, and all are scored as yes or no. The number of yes scores of the respective index are summed to generate its total score. Given the HOME Inventory is a formative measure [26], we used the eight sub-scales as independent indices.

The HOME-MC was adapted prior to use in the study following a step-by-step process. The items were reviewed by the local team members residing in the same district with experiences of collecting data or coordinating previous follow-ups with neurodevelopmental measures and a senior investigator. During the review, two items were deemed inappropriate for the context under the Enrichment index (*Family provides lessons or organizational membership to support child’s talents membership*,* gymnastic lessons*,* art center*, etc. and *Child has access to a library card*,* and family arranges for child to go to library once a month*) as no libraries or other organizational membership opportunities exist in the setting. No alternatives were possible either. Hence, these items were dropped from the final tool. Next, the form and guidelines for assessors were created in local language. Translation and back translations were carried out for quality checks. The form was piloted in two cycles with 5 mothers (comprising those who can read and who cannot). Feedback was sought from the assessors about the items, time taken and the user-friendliness of the form before finalization.

### Fluid reasoning abilities

The Wechsler Intelligence Scales for Children-5th edition (WISC -V) [34], also developed in the US, is one of the most widely used tests for general abilities. For the current study, we used the Fluid Reasoning Index (FRI) composed of two performance indices: Matrix Reasoning and Figure Weights. These indices are non-verbal and deemed to be relatively culturally fair. A study across seven countries examining the effect of stunting on cognitive development for children 5 years of age, used the fluid reasoning index of the Wechsler Scales of Preschool and Primary Scale of Intelligence-3rd edition and found it to hold adequate psychometric properties [35]. For analysis, we used US norms with mean of 100 and standard deviation of 15 [34].

### Sociodemographic characteristics

Standard interview questionnaires were used to collect data on socioeconomic and demographic characteristics of the study sample including maternal and paternal education and occupation, child school enrolment and number of household members. These questionnaires have also been used in the previous follow-up of the cohort [28, 29].

## Data collection procedures

Data was collected by a trained and experienced team of research assistants (RAs) from June to November 2017. Subjects were identified, screened, consented and enrolled at home followed by administration of the data collection measures. Some of the RAs had been working in this role for more than 8 years, collecting data related to child development (including HOME inventory and intelligence scales) for different projects. They were trained by a senior supervisor who had similarly been in the role for about 8 years. The training for each measure entailed 3 days in the classroom followed by field practice for two weeks. Each RA was certified after an inter-rater agreement of at least 80% with the supervisor.

The HOME-MC was administered at the child’s home. Some items were scored after observation by the RA and some as an interview of the primary caregiver as per the HOME-MC guidelines provided in the manual (Refer to Appendix Table S1 for list of items and mode of administration).

For administration of the WISC V, a dedicated space (with minimal distractions) in each village was temporarily set up during the data collection period. Child and a caregiver were provided pick and drop to the space. For quality assurance, 10% of the assessments were scored for inter-reliability scores by the supervisor covering all the RAs. Intra-class coefficient scores for inter-rater agreement (*N* = 125) ranged from 0.91 to 0.95 on the different indices. The Principal Investigator (first author) made monthly visits to the field site to support training, provide supervision and to monitor data collection.

### Data analysis

The analysis was conducted using Stata version 16. We computed the mean (standard deviation, SD) and median (interquartile range, IQR) of the HOME indices. The descriptive analysis of the WISC FRI has also been published in another paper [36]. Raw total scores on the HOME-MC indices were converted to z-scores to facilitate comparison between the indices. The R^2^ was extracted from single and multivariable regression models with the eight index scores as exposure and FRI scores as the outcome to explore the association as per the study objective. We first assessed the bivariate association between each index and the FRI scores to document the crude association of each index. Next, all were entered simultaneously as predictors to the FRI scores to assess multivariate associations. From the multiple regression analysis, we also report the semi-partial correlations squared (*sr*^2^) which is interpreted as the proportion of the total variance in the dependent variable that is uniquely explained by each index. We also tested the association per item within each index of the HOME-MC to examine which items were driving the association.

Given the role of maternal capacities in home environment and child’s cognitive skills, we also adjusted the multivariable analysis for maternal education, measured as number of grades completed. We also adjusted the item-wise associations of HOME items with FRI for maternal education. Since school enrolment can influence intellectual abilities, we carried out subgroup analyses based on school enrolment status.

## Results

Sociodemographic characteristics of the sample are presented in Table 1. A total of 1172 caregivers were administered the HOME-MC Inventory. The mean age of the child was 7.9 years at the time of assessment. The percentage of girls (45.4) was slightly lower in the cohort compared to boys (54.5). Only 503 children (43%) of the sample were enrolled in school, with the majority enrolled in grade 1 (281, 23.9%) followed by grade 2 (179, 14.8%). About two-thirds of the mothers and one-third of the fathers were not able to read and write. Living in extended families was common (67.8%) with an average of 10 household members in the house.

**Table 1:** Sociodemographic characteristics of the study participants (*N* = 1172)

Variable	*N* (%)
Child age in years (Mean, SD)	7.9 (0.26)
Child sex Female Male	531 (45.3) 641 (54.7)
Enrolled in school	503 (42.9)
Type of school Government Private NGO	337 (28.8) 154 (13.1) 12 (1.0)
Current grade 1 2 3 4	281 (23.9) 179 (15.3) 32 (2.7) 3 (0.3)
Maternal education in no. of years None Primary (1–5) Above primary (> 5)	761 (64.9) 246 (20.9) 165 (14.2)
Maternal employment*	302 (25.7)
Paternal education in no. of years None Primary (1–5) Primary to metric (6–10) Above metric (> 10)	371 (31.7) 141 (12.0) 342 (29.2) 318 (27.1)
Paternal occupation Unemployed Labourer Professional Agriculture Small business Handicraft	37 (3.3) 571 (50.1) 221 (19.6) 154 (13.6) 119 (10.5) 29 (2.6)
Type of family Nuclear Joint/extended	377 (32.2) 795 (67.8)
Total family members (Mean, SD)	10.5 (5.7)
Total children up to 15 years (Mean, SD)	5.2 (2.8)

Note Data is presented as N (%) unless stated otherwise. NGO = nongovernment organization

*Maternal employment included teaching or a household worker on daily wages

Table 2 presents descriptive statistics of the eight HOME-MC indices. On Responsivity, the mean was 6.7 (SD 1.6) and the range of 2–10 shows no family scored 0 on this index. However, on six indices including Encouragement, Emotional climate, Learning Opportunities, Enrichment, Family companionship, and Physical environment the lowest value was 0. The lowest scores were for Learning opportunities with a mean of 2.4 (SD 1.1), with a maximum possible score of 8.

**Table 2:** Descriptive data of the HOME-MC indices (*N* = 1172)

Index	Mean (SD)	Median (IQR)	Range	Max possible
Responsivity	6.7 (1.6)	7 (6,8)	2,10	10
Encouragement	4.5 (1.4)	5 (4,5)	0,7	7
Emotional climate	3.9 (1.6)	4 (3,5)	0,8	8
Learning materials & opportunities	2.4 (1.1)	2 (2,3)	0,7	8
Enrichment	2.1 (1.2)	2 (1,3)	0,6	6
Family companionship	4.5 (1.1)	5 (4,5)	0,6	6
Family integration	3.3 (0.6)	3 (3,4)	1,4	4
Physical environment	4.0 (1.7)	4 (3,5)	0,8	8

Bivariate analyses revealed that all indices except for Family enrichment were significantly associated with FRI. Multivariable linear regression analyses measuring the associations between converted z-scores of the HOME-MC and the FRI, revealed that Learning materials and opportunities had the highest coefficient (β = 1.74, 95% CI = 1.15, 2.34) followed by Responsivity (β = 1.73, 95% CI = 1.07, 2.38) and Family companionship (β = 1.27, 95% CI = 0.63, 1.90) (Table 3). The eight different indices of the HOME-MC collectively explained 22% of the total variation in FRI scores, with Learning materials and opportunities (*sr*^2^ of 0.022), Responsivity (*sr*^2^ of 0.17) and Family companionship (*sr*^2^ of 0.10) had the highest unique explained variance of the FRI.

**Table 3:** Associations between HOME-MC indices and the WISC V FRI scores (*N* = 1172)

Index	Bivariate analysis				Multivariable analysis Adjusted *R*^2^ = 0.22			
	β	95% CI	*R* ^2^	*p*-value	β	95% CI	*p*-value	sr^2^
Responsivity	3.71	3.15, 4.25	0.13	< 0.001	1.73	1.07, 2.38	< 0.0001	0.017
Encouragement	3.28	2.72, 3.85	0.10	< 0.0001	0.91	0.27, 1.52	0.005	0.005
Emotional climate	2.16	1.58, 2.74	0.04	< 0.0001	0.52	− 0.03, 1.08	0.068	0.002
Learning materials & opportunities	3.49	2.93, 4.05	0.11	< 0.0001	1.74	1.15, 2.34	< 0.0001	0.022
Enrichment	3.32	2.76, 3.88	0.10	< 0.0001	0.85	0.18, 1.52	0.013	0.004
Family companionship	3.33	2.77, 3.89	0.10	< 0.0001	1.27	0.63, 1.90	< 0.0001	0.010
Family integration	0.15	− 0.44, 7.41	0.00	0.621	-0.47	-1.01, 0.06	0.085	0.002
Physical environment	1.90	1.31, 2.48	0.03	< 0.0001	0.43	− 0.11, 0.98	0.121	0.002

Note: β = beta coefficient, CI = confidence intervals, sr^2 =^ semi-partial correlations squared

In prediction models by school enrolment status, the HOME-MC indices contributed to 13% variation in the FRI score in children who were enrolled and 16% for those who were not enrolled with slightly higher coefficients for the enrolled sample on Responsivity (β = 1.85, 95% CI = 0.77, 2.92 vs. β = 1.07, 95% CI = 0.34, 1.79) and Learning Opportunities (β = 1.54, 95% CI = 0.62, 2.45 vs. β = 1.24, 95% CI = 0.53, 1.95) (Supplementary Table 1).

Adjusting for maternal education modestly increased the R^2^ to 0.26 (up from 0.22 on unadjusted). The strength of the associations between FRI scores and indices were slightly reduced, though the pattern of results remained essentially the same (Supplementary Table S2). This is visually represented in a figure with the item-wise associations of HOME items with FRI scores (Supplementary Figure S1).

**Fig. 1:**
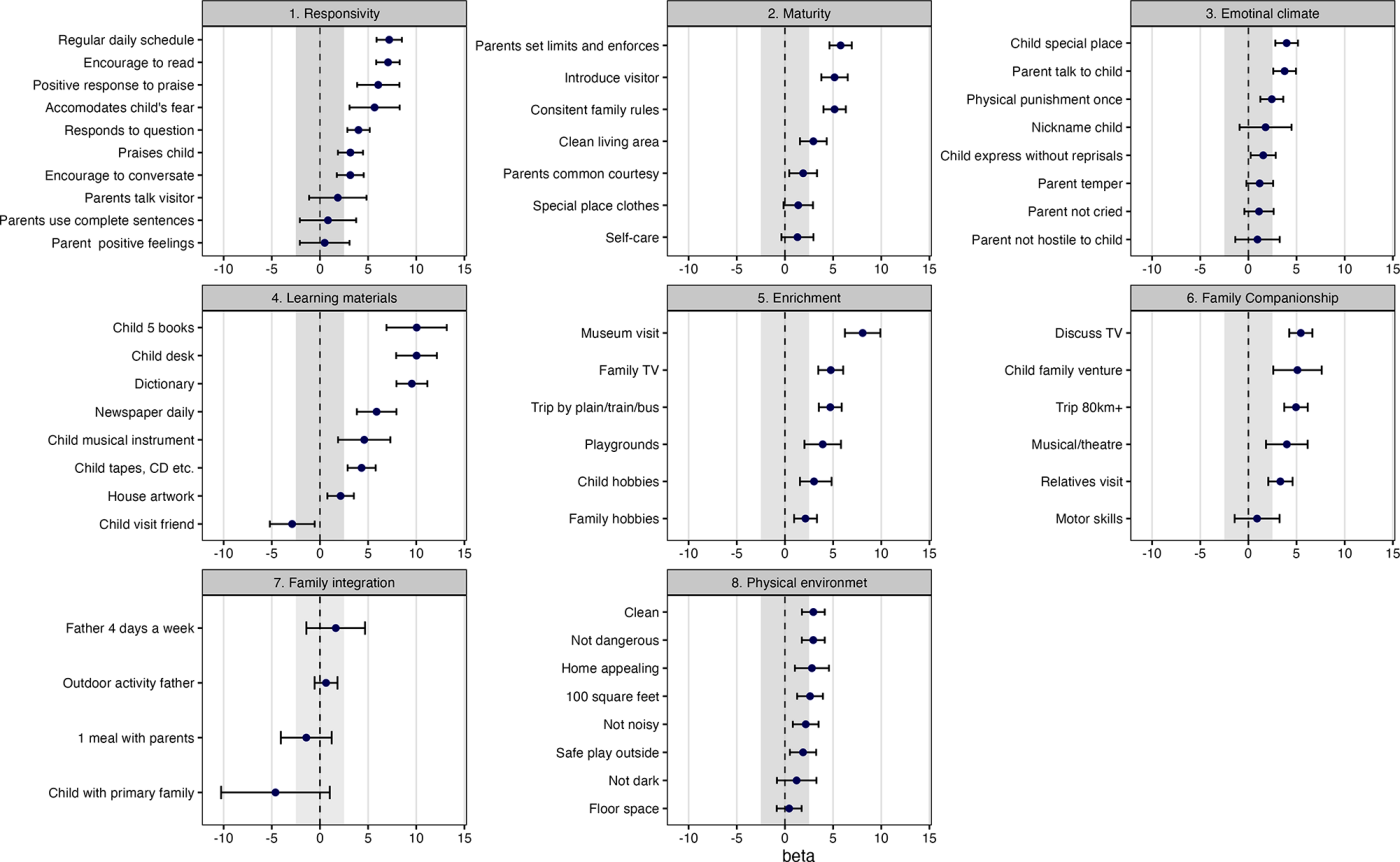
Item-wise regression of HOME indices with FRI scores

Examination of the variation per each item of the HOME-MC indices revealed variation on Responsivity and Learning materials and opportunities indices but in different directions. On responsivity 5 of 10 items were scored as ‘yes’ by more than 90% of participants while on the latter 5 of 8 items were scored as ‘no’ by more than 85% of the participants (Supplementary Table 2). Item-wise regression analyses with the WISC FRI scores are shown in Fig. 1. Items assessing access to learning materials and the emphasis by families on education e.g., the child having access to 5 books (β = 10.04, 95% CI = 6.91, 13.17), or a desk (β = 10.03, 95% CI = 7.91, 12.14) or dictionary (β = 9.54, 95% CI = 7.94, 11.15) had strongest observable associations. On the family companionship index, discussing TV show with child had the highest coefficient (β = 5.44, 95% CI = 4.24, 6.64) and having a regular and predictable schedule (β = 7.19, 95% CI = 5.88, 8.51) on the responsivity index, had the highest coefficients.

## Discussion

The objective of the study was to identify the features of home environment as measured by HOME -MC scales associated with fluid reasoning abilities as indicated FRI scores of WISC-V and determine to what extent these features influence those abilities in middle childhood in a high-risk setting in Pakistan. The findings from the multiple regression analysis revealed that the indices with strongest association were Learning materials and opportunities, Responsivity and Family companionship. No association was found with Family integration and Physical environment. Item-wise examination of associations revealed items within each index driving the strength of the associations e.g., on the Learning materials it was child having access to 5 books and having a predictable schedule on the Responsivity index.

Learning materials and opportunities and Responsivity have been found to be critical for the early years in disadvantaged contexts in LMIC [1–4] and the current study found that their influence continues in middle childhood. The items on the learning materials and opportunities index assess the child’s access to stimulative items. The items with the highest coefficient were access to at least 5 books and to a desk/learning space. Some of these items could indicate the value parents lay on a child’s learning. Similar findings have been reported from studies evaluating children’s academic achievement. A meta-analysis of the effects of home literacy environment on children’s reading environment found that parental involvement and literacy expectations of children had significantly higher association contributed to child’s literacy knowledge than availability of literacy resources [37].

Of the items on the Responsivity index, the ones with the strongest association were ‘Child is encouraged to read on his own’ and ‘Family has a fairly predictable schedule’. The difference between the two indices i.e., Learning material and Responsivity with respect to variation of items could be explained by the fact that access to learning materials indicates more of community development with availability in the local market and a functional education system that would encourage the use of such resources as compared to being responsive within one’s environment. Hence, our findings suggest that intervention studies intending to increase parental use of resources will need to be designed to keep the larger picture of the community in perspective.

Another index of the HOME-MC found to be significant was Family companionship as defined by child involvement with the parents in activities providing companionship as a source of mutual enjoyment in everyday life such as visiting family or going out. These mutually enjoyable experiences with family can also be seen as learning opportunities, exposing the child to different settings with potential parental guidance that contributes to the child’s intellectual abilities. However, though the item measures mutual feelings, the answer is reported by the parent and perhaps reflects the parental perspective more than that of the child. Given the cultural context, it is likely that fathers were involved with outdoor activities [38, 39]. Paternal involvement and time spent with children have also been shown to benefit children’s intellectual abilities [8]. We realize that these factors may also reflect the socioeconomic advantage of the families who can afford such trips and ventures. Nonetheless, even in highly disadvantaged settings, such opportunities are of value for children’s development. Research also suggests that shared family times is an important factor for family resilience which has been associated with positive outcomes for children [40].

Encouragement of maturity and Enrichment were also found to be associated with the FRI scores which aligns with the literature. A meta-analysis examining the effect of parenting behaviours and executive functioning, found significant associations between parental behaviours that promote autonomy, scaffolding and stimulation and child executive functioning in children between 0 and 8 years [41]. Another study in the US with 8–12 year old children found that enrichment activities as measured with HOME, mediated the relationship between family socioeconomic status and child outcomes [42].

The indices Emotional climate, Family integration, and Physical environment were not associated with the WISC FRI scores. This could be due to differences in sociocultural context, and that family integration in a rural Pakistani context may not be the same as in a disadvantaged context in the US (country of origin of the tool). Most of the items in this index assess activities with the father or father figure for example, which had a very low variation in the current study context. Moreover, in this setting children enjoy frequent interactions with extended family members which is not captured in the HOME inventory. Findings could suggest that the adaption process of the inventory ahead of study start was not sufficient. In future studies, a different approach for adaptation needs to be taken, for instance like the one described by Holding et al. [43], who conducted focus group discussions with families to identify constructs and develop a pool of items that could serve as indicators.

Given the association of the home environment for fluid reasoning skills, specifically learning materials, and parental responsivity and engagement in the current study, it highlights the need for future longitudinal cohort studies to better establish the temporal order of the associations found between home and fluid reasoning indices. Also, it is an important aspect to consider for long-term benefits of the impact of interventions on child development outcomes, as they tend to fade over time [44]. Interventions for children in this age group have mostly focused on school-based interventions [6] but may benefit from engaging parents to improve home environment for learning.

The study has several strengths, including high-quality data using standardised measures of home environment and child fluid reasoning abilities, from a highly disadvantaged context posing multiple risks in middle childhood when children start primary education, and a large community sample size. The study also has limitations. Inclusion of the Verbal scale and the Full-Scale IQ of the WISC-V would have strengthened the study, but we were limited due to the logistics of administering the full scale to derive these indices. Moreover, two items were removed from the Enrichment subscale of the HOME-MC as they were considered not suitable for the current cultural setting. Although this may slightly impact the meaning of the Enrichment subscale, and thus also the comparability of our results with other studies, it was deemed important to ensure the scale’s relevance and appropriateness for this specific cultural context. Another potential source of inaccuracy might be that items related to activities outside the home could be better addressed by fathers or male caregivers, considering the sociocultural context.

We conclude that the relationship between features of the home environment and fluid reasoning abilities during middle childhood in disadvantaged settings is comparable to the association between home environment and cognitive development in early childhood years. Given this significance, more attention in Global ECD research is needed to study the influence of the home environment during middle childhood on child outcomes.

## Electronic supplementary material

Below is the link to the electronic supplementary material.

Supplementary Material 1

Supplementary Material 2

## Data Availability

No datasets were generated or analysed during the current study.
