# Supplementary material for: Is home environment associated with child fluid reasoning abilities in middle childhood in high-risk settings? findings from a cross-sectional study in Pakistan
Source: BMC Pediatr. 2024 Oct 8;24:638. doi: 10.1186/s12887-024-05108-z (PMC11459995; doi:10.1186/s12887-024-05108-z)
Supplement: Supplementary file 1 — Supplementary Material 1 [file 12887_2024_5108_MOESM1_ESM.docx]

Supplementary Table 1

N (%) of ‘Yes’ responses on HOME-MC items

| **S No.** | **I. RESPONSIVITY** | Yes N (%) |
| --- | --- | --- |
| 1. | Family has fairly regular & predictable daily schedule for child (mealtime, school / madrassa time, bedtime TV time, homework time, etc.). I | 298 (23.5) |
| 2. | If child has a fear (e.g., afraid of the dark), then parent follows all of the child’s requests (e.g., keeping the light on all night when the child is sleeping) **I** | 1202 (94.8) |
| 3. | Child has been praised at least twice during past week for doing something. **I** | 929 (73.3) |
| 4. | Child is encouraged to read on his own. (e.g., story books or magazines). **I** | 384 (30.3) |
| 5. | Parent encourages child to contribute to the conversation during visit. (e.g., Greet the aunty, ask aunty how she is?) **O** | 290 (22.8) |
| 6. | Parent shows some positive emotional response to praise of child by Visitor. **O** | 1174 (92.6) |
| 7. | Parent responds to child’s questions during visit. **O** | 598 (22.7) |
| 8. | Parent uses complete sentence structure and some long words in conversing. **O** | 1214 (95.8) |
| 9. | When speaking of or to child, parent’s voice conveys positive feelings. **O** | 1197 (94.5) |
| 10. | Parent initiates verbal interchanges with Visitor, asks questions, makes spontaneous comments. **O** | 1219 (96.2) |
|  | **II. ENCOURAGEMENT OF MATURITY** |  |
| 11. | Family requires child to carry out certain self-care routines, e.g., makes bed, cleans room, cleans up after spills, bathes self. **I** | 1076 (84.9) |
| 12. | Family requires child to keep living and play area reasonably clean and straight. **I** | 977 (77.1) |
| 13. | Child puts own school uniform, dirty clothes and other regular clothes in special place **I** | 1031 (81.3) |
| 14. | Parents set limits for child and generally enforce them (e.g., finish homework before TV, sleep time). **I** | 770 (60.7) |
| 15. | Parent is consistent in establishing or applying family rules. **I** | 526 (41.5) |
| 16. | Parent introduces Visitor to child. **O** | 284 (22.4) |
| 17. | Parents follow the rules of common courtesy (e.g., Saying Salam and offer seat, ask for tea). **O** | 1011 (79.8) |
|  | **III. EMOTIONAL CLIMATE** |  |
| 18. | Parent has not lost temper with child more than once during previous week. **I** | 299 (23.6) |
| 19. | Parent reports no more than one instance of physical punishment occurred during past month. **I** | 564 (43.1) |
| 20. | Child can express negative feelings toward parents without harsh reprisals. **I** | 365 (28.8) |
| 21. | Parent has not cried or been visibly upset in child’s presence more than once during past week, expect in case of death in family. **I** | 1034 (81.6) |
| 22. | Child has a special place in which to keep his/her possessions. **E** | 683 (53.9) |
| 23. | Parent talks to child during visit (beyond correction and introduction). **O** | 741 (58.5) |
| 24. | Parent uses a term of affection or a nick name for the child (e.g. *My jaan, jano,bablo)*! at least twice during visit. **O** | 63 (4.9) |
| 25. | Parent does not express overt annoyance with or hostility toward child (complains, describes child as “bad,” says child won’t mind, etc.). **O** | 1181 (93.2) |
|  | **IV. LEARNING MATERIALS & OPPORTUNITIES** |  |
| 26. | Parent buys and reads a newspaper daily. **I** | 114 (9) |
| 27. | Family has a dictionary and encourages child to use it. **I** | 182 (14.3) |
| 28. | Child has visited a friend by him/herself in the past week. **I** | 1183 (93.4) |
| 29. | Child has free access to tapes, CD, or record player or radio or mobile phone. **I** | 1024 (80.8) |
| 30. | Child has free access to real/toy musical instrument (piano, drum, ukulele, or guitar, etc.). **E** | 67 (5.3) |
| 31. | Child has free access to at least 5 appropriate books (excluding textbooks and *Quran Majeed)*. **E.** | 44 (3.5) |
| 32. | Child has free access to desk or another suitable place for reading or studying. **I** | 98 (7.7) |
| 33. | House has at least two pictures or other type of artwork on the walls. **O** | 310 (24.5) |
|  | **V. ENRICHMENT** |  |
| 34. | Family has a TV, and it is used judiciously, not left on continuously. **I** | 338 (26.7) |
| 35. | Family encourages child to develop or sustain hobbies. **I** | 1016 (80.2) |
| 36. | Child is regularly included in family’s recreational hobby. **I** | 556 (43.9) |
| 37. | Child has ready access to at least two pieces of playground equipment in the immediate vicinity. **(**e.g., swings, climbing bars, volleyball net cricket pitch). **I** | 128 (10.1) |
| 38. | Family member has taken child to (or arranged for child to visit) a scientific, historical or art museum within the past year. **I** | 135 (10.6) |
| 39. | Family member has taken child on (or arranged for child to take) a plane, train, or bus trip within the past year. **I** | 477 (37.7) |
|  | **VI. FAMILY COMPANIONSHIP** |  |
| 40. | Family visits or receives visits from relatives or friends at least twice a month. **I** | 889 (70.2) |
| 41. | Child has accompanied parent on a family business venture 3-4 times within the past year (to garage, clothing shop, appliance repair shop, etc.). **I** | 1193 (94.2) |
| 42. | Family member has taken child, (or arranged for child to attend) some type of live musical or theatre performance. **I** | 1163 (91.8) |
| 43. | Family member has taken child on (or arranged for child to take) a trip of more than 80km from home (80km radial distance, not total distance). **I** | 418 (32.9) |
| 44. | Parents discuss TV programs with child. **I** | 802 (63.3) |
| 45. | Parent helps child to achieve advance motor skills ride a two-wheel bicycle, roller skate, play ball, etc. **I** | 1182 (93.3) |
|  | **VII. FAMILY INTEGRATION** |  |
| 46. | Father (or father substitute) regularly engages in outdoor recreation with child (e.g. fishing, hunting or gardening). **I** | 540 (42.6) |
| 47. | Child sees and spends some time with father or father figure 4 days a week. **I** | 1215 (95.9) |
| 48. | Child eats at least 1 meal per day, on most days, with mother and father (or mother and father figures). **I** | 1200 (94.7) |
| 49. | Child has remained with this primary family group for all his life aside from 2-3 week vacations, illnesses of mother, visits to grandparents, etc. **I** | 1254 (98.9) |
|  | **VIII. PHYSICAL ENVIRONMENT** |  |
| 50. | Home has a picture or wall decoration appealing to children (e.g pictures of cartoons, numbers, alphabets etc). E | 159 (12.5) |
| 51. | The interior of the home or apartment is not dark or perceptually monotonous. **O** | 1158 (91.4) |
| 52. | In terms of available floor space, the rooms are not overcrowded with furniture. **O** | 896 (70.7) |
| 53. | All visible rooms of the house are reasonably clean and minimally cluttered. **O** | 725 (57.2) |
| 54. | There is at least 100 square feet of living space per person in the house including courtyard. **O** | 326 (25.7) |
| 55. | House is not overly noisy—TV, shouts of children, radio, etc. **O** | 944 (74.5) |
| 56. | Building has no potentially dangerous structural or health defects (e.g., plaster coming down from ceiling, stairway boards missing, rodents, etc.). **O** | 531 (41.9) |
| 57. | Child’s outside play environment appears safe and free of hazards. (No outside play area requires an automatic minus.) **E** | 333 (26.3) |

Note: HOME-MC= Home Environment for Measurement of Environment-Middle Childhood

Supplementary Table 2

Associations between HOME-MC indices and the WISC V FRI scores adjusted for maternal education (N=1172)

| Subscale | Multivariable analysis adjusted*  Adjusted R^2^= .26 | | | | |
| --- | --- | --- | --- | --- | --- |
|  | β | 95% CI | p-value | *sr^2^* |  |
| Responsivity | 1.50 | .85, 2.15 | .000 | .013 |  |
| Encouragement | .87 | .25, 1.49 | .006 | .004 |  |
| Emotional climate | .46 | -.08, 1.02 | .100 | .001 |  |
| Learning materials & opportunities | 1.30 | .71, 1.89 | .000 | .011 |  |
| Enrichment | .59 | -.08, 1.26 | .086 | .001 |  |
| Family companionship | 1.20 | .58, 1.82 | .000 | .009 |  |
| Family integration | -.42 | -.95, .11 | .120 | .002 |  |
| Physical environment | .28 | -.25, .83 | .295 | .000 |  |

Note: β = beta coefficient, CI = confidence intervals, sr^2 =^ semi-partial correlations squared

*Adjusted for maternal education

Supplementary Table 3

Associations between HOME-MC components Iand the WISC V FRI scores by child school enrolment status (N=1172)

| Subscale | Enrolled  Multivariable analysis  Adjusted R^2^=.12 | | | Not enrolled  Multivariable analysis  Adjusted R^2^ = .16 | | |
| --- | --- | --- | --- | --- | --- | --- |
|  | coef. | 95% CI | p-value | coef. | 95% CI | p-value |
| Responsivity | 1.85 | .77, 2.92 | .001 | 1.07 | .34, 1.79 | .004 |
| Encouragement | -.19 | -1.27, .89 | .729 | 1.12 | .43, 1.79 | .001 |
| Emotional climate | 1.01 | .09, 1.92 | .031 | -.03 | -.67, .60 | .922 |
| Learning opportunities | 1.54 | .62, 2.45 | .001 | 1.24 | .53, 1.95 | .001 |
| Enrichment | .38 | -.65, 1.42 | .469 | .77 | -.03, 1.58 | .060 |
| Family companionship | .93 | -.18, 2.04 | .102 | 1.21 | .54, 1.88 | .000 |
| Family integration | .21 | -.88, .94 | .638 | -.62 | -1.24, -.00 | .050 |
| Physical environment | .03 | -.88, .94 | .949 | .03 | -.58, .64 | .912 |
