# Supplementary figures and images for: Is home environment associated with child fluid reasoning abilities in middle childhood in high-risk settings? findings from a cross-sectional study in Pakistan
Source: BMC Pediatr. 2024 Oct 8;24:638. doi: 10.1186/s12887-024-05108-z (PMC11459995; doi:10.1186/s12887-024-05108-z)

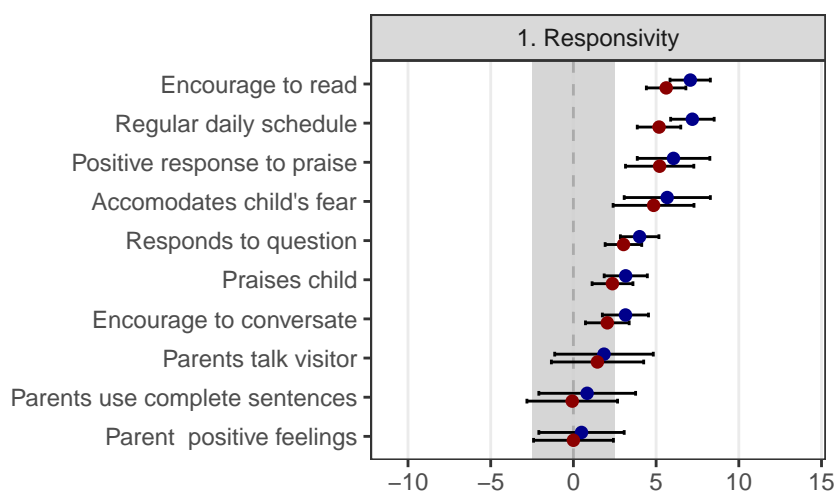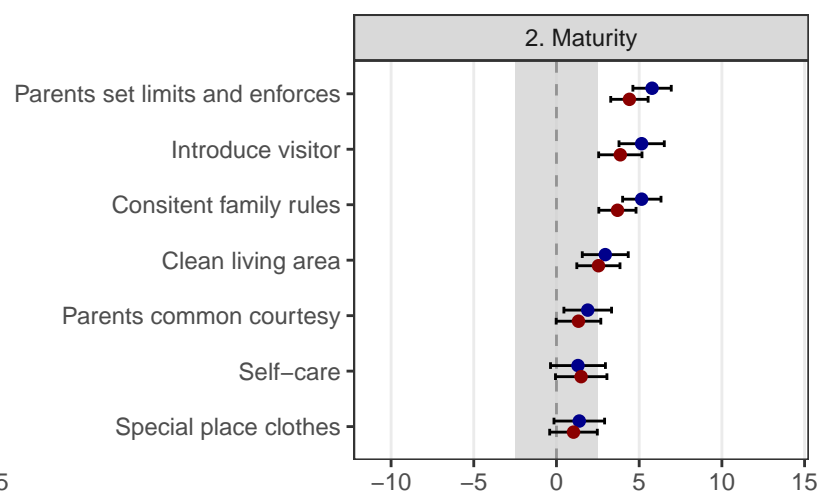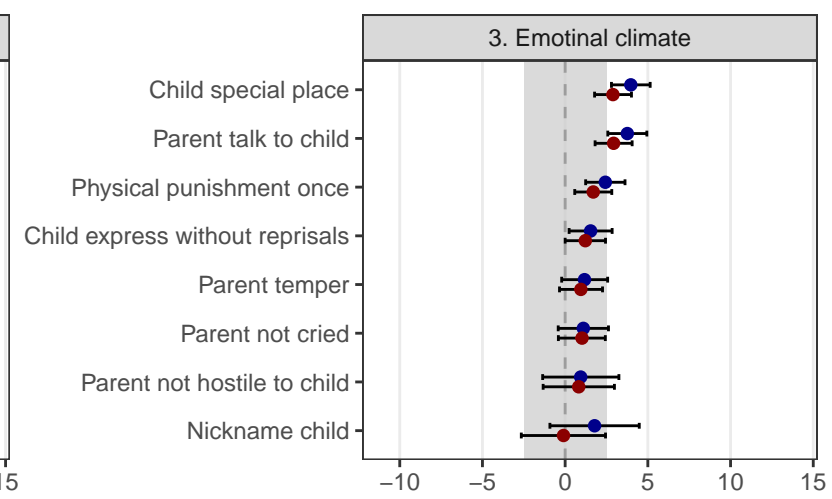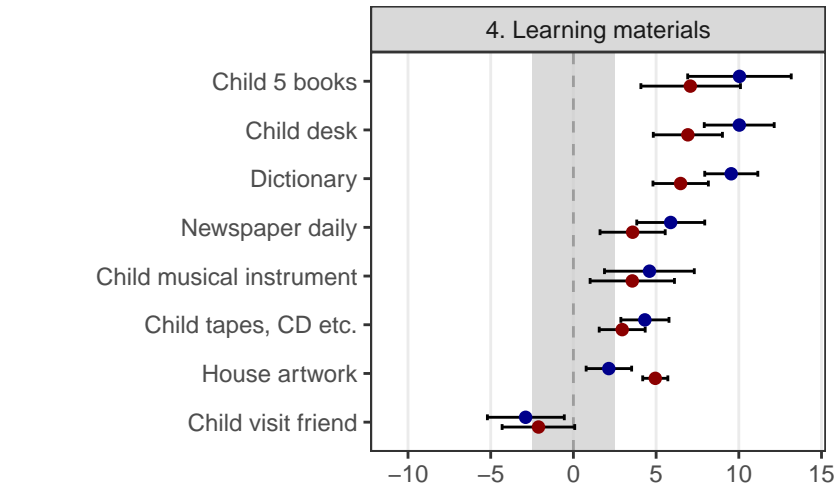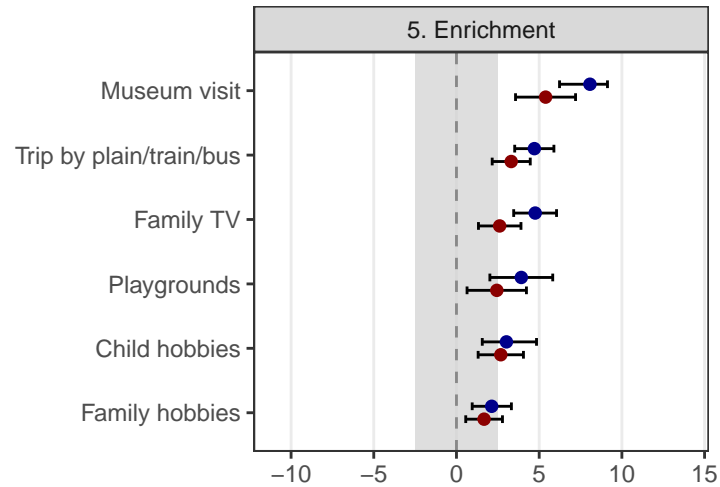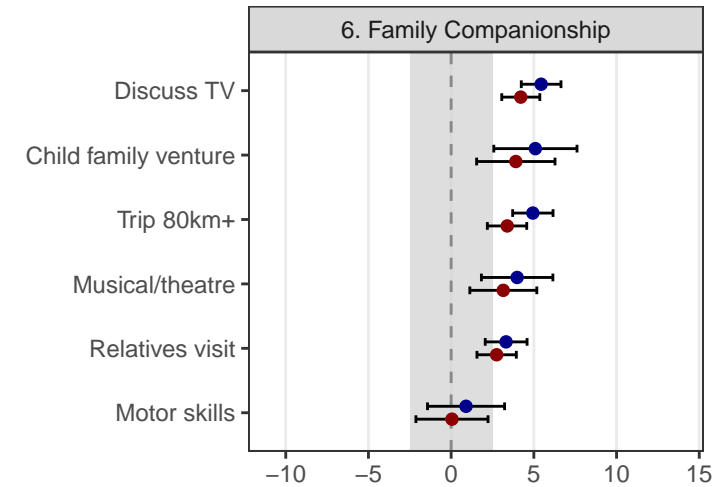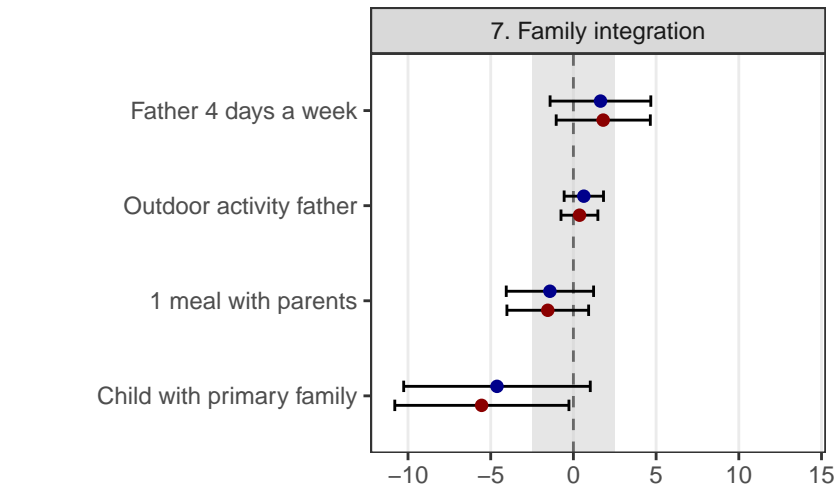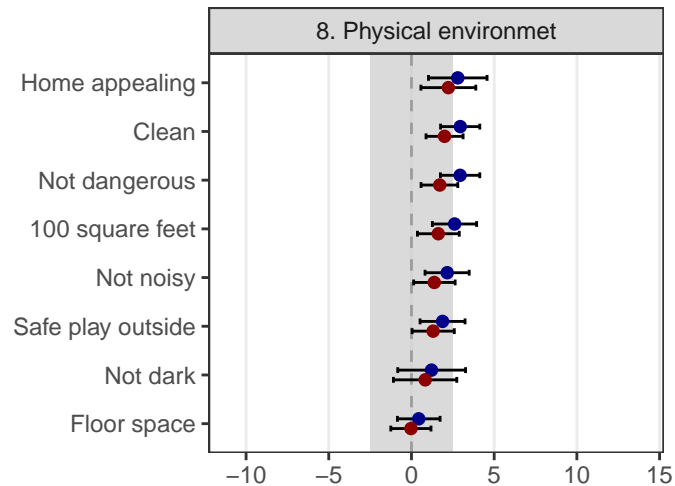

Model

- Crude
- Adjusted

Supplement: Supplementary file 2 — Supplementary Material 2 [file 12887_2024_5108_MOESM2_ESM.pdf]
